# Breast Cancer Treatment Using the Magneto-Hyperthermia Technique Associated with Omega-3 Polyunsaturated Fatty Acids’ Supplementation and Physical Training

**DOI:** 10.3390/pharmaceutics16030310

**Published:** 2024-02-22

**Authors:** Matheus Haubert Theinel, Mariana Penteado Nucci, Gabriela Cianciarullo, Fernando Anselmo Oliveira, Arielly da Hora Alves, Javier Bustamante Mamani, Gabriel Nery de Albuquerque Rego, Nicole Mastandrea Ennes do Valle, Olívia Furiama Metropolo Dias, Cícero Júlio Silva Costa, Felipe Lima Nascimento, Juan Matheus Munoz, Andressa Arruda de Moraes, Lionel Fernel Gamarra

**Affiliations:** 1 Hospital Israelita Albert Einstein, São Paulo 05652-000, SP, Brazil; nutrimatheusht@gmail.com (M.H.T.); gabi.ciancia@gmail.com (G.C.); fernando.ao@einstein.br (F.A.O.); ariellydahora1997@gmail.com (A.d.H.A.); javier.mamani@einstein.br (J.B.M.); gabriel.nery@einstein.br (G.N.d.A.R.); nicolemev@gmail.com (N.M.E.d.V.); olivia.metropolo@einstein.br (O.F.M.D.); cicero.costa@einstein.br (C.J.S.C.); felipe.lnascimento@einstein.br (F.L.N.); juanmunoz@usp.br (J.M.M.); andressaarrudamoraes@gmail.com (A.A.d.M.); 2 LIM44—Hospital das Clínicas da Faculdade Medicina da Universidade de São Paulo, São Paulo 05403-000, SP, Brazil; mariana.nucci@hc.fm.usp.br

**Keywords:** omega-3, breast cancer, polyunsaturated fatty, bioluminescence imaging, magnetic-hyperthermia, iron oxide nanoparticles, cancer treatment, physical exercise

## Abstract

Breast cancer (BC) presents a growing global concern, mainly for the female population of working age. Their pathophysiology shows challenges when attempting to ensure conventional treatment efficacy without adverse effects. This study aimed to evaluate the efficacy of magneto-hyperthermia (MHT) therapy associated with supplementation with omega-3 polyunsaturated fatty acid (w-3 PUFA) and engagement in physical training (PT) for the triple-negative BC (TNBC) model. First, we assessed the physicochemical properties of iron oxide nanoparticles (ION) in biological conditions, as well as their heating potential for MHT therapy. Then, a bioluminescence (BLI) evaluation of the best tumor growth conditions in the TNBC model (the quantity of implanted cells and time), as well as the efficacy of MHT therapy (5 consecutive days) associated with the previous administration of 8 weeks of w-3 PUFA and PT, was carried out. The results showed the good stability and potential of ION for MHT using 300 Gauss and 420 kHz. In the TNBC model, adequate tumor growth was observed after 14 days of 2 × 10^6^ cells implantation by BLI. There was a delay in tumor growth in animals that received w-3 and PT and a significant decrease associated with MHT. This pioneering combination therapy approach (MHT, omega-3, and exercise) showed a positive effect on TNBC tumor reduction and demonstrated promise for pre-clinical and clinical studies in the future.

## 1. Introduction

Breast cancer (BC) is the most common cancer worldwide. In 2020, there were 2.3 million new diagnoses of BC, and 685,000 people died worldwide. At the end of 2020, 7.8 million living women had received a diagnosis in the past five years [1]. Aging, family history, genetics, early menarche, late menopause, advanced age at first pregnancy, low parity, endogenous and exogenous estrogen levels, and lifestyle habits are all associated with the development of BC in women [2]. Tumor growth, invasiveness, proliferation, and gene expression are among the characteristics [3,4,5,6] that distinguish the primary subtypes of BC, which include Luminal A, which accounts for roughly 40–50% of cases, Luminal B, which is estimated to account for 20–30% of cases, HER2+, which accounts for 15–20% of cases, and triple-negative breast cancer (TNBC), which accounts for 10–20% of cases [7,8,9]. This subtype analysis is essential to ensure a correct diagnosis and effective treatment [6,9,10].

Treatment for BC often consists of local (mastectomy and radiation) as well as systemic (chemotherapy, hormone therapy, and immunotherapy) therapies [11]. Nevertheless, the most widely utilized therapies have negative impacts on mastectomized women’s quality of life and body image, in addition to producing toxicity via the use of radiation or antineoplastic drugs [12,13]. However, other promising therapeutic approaches are emerging, such as localized therapy of the tumor with the application of magnetic nanoparticles (NP) in the presence of an alternating magnetic field. This therapy, known as magneto-hyperthermia (MHT), involves either directly administering NP into the tumor or functionalized magnetic NP when exposed to an alternating magnetic field (AMF) for a predetermined amount of time at a frequency with amplitudes between 10 and 30 kA/m. Following this, the temperature raises at the tumor site to 40 to 43 °C via magnetic relaxation mechanisms (Brownian and Neel relaxation) [14,15]. This temperature change affects the physiology of tumor cells, inducing apoptosis and/or cellular necrosis [15,16,17]. The MHT technique has its advantages as a non-invasive approach, as it is applicable to deep-seated tumors, causes the selective death of tumor cells without harming surrounding healthy tissues, and has a high heating performance compared to other heat-based therapies [18,19], showing its efficacy in tumor therapeutics [20]. One of the most magnetic NP used in this technique is iron oxide nanoparticles (ION), which have been used in a series of preclinical studies to treat various tumors [21,22,23,24,25,26], as well as BC [27,28].

Supplementation with omega-3 polyunsaturated fatty acids (PUFAs) has been investigated as an additional therapeutic approach in pre-clinical [29,30,31] and clinical [32,33] research on BC treatment. Several studies have confirmed the benefits of utilizing omega-3 PUFAs in cancer prevention, while the same was not observed for omega-6 PUFAs [34]. Omega-3 PUFAs have antioxidant and anti-inflammatory properties [35,36], contributing to the reduction in cytokine production, cell proliferation, and, consequently, tumor growth, leading to an apoptosis of tumor cells [37]. Another remarkable component of the prevention and treatment of BC is physical exercise. There is scientific evidence of the significant benefits that patients at different stages of the disease can receive from regular exercise [38,39,40], and it has also been associated with the consumption of omega-3 PUFAs and healthy lifestyle habits [41,42,43].

Thus, this study aimed to evaluate the efficacy of MHT therapy associated with supplementation with omega-3 PUFA and engaging in physical training for the TNBC model.

## 2. Materials and Methods

### 2.1. ION Physicalchemical Evaluation

#### 2.1.1. ION Characterization

In this study, we used commercial IONs (fluidMAG-Q) purchased from the company Chemicell (Chemicell, FluidMAG-Q, Berlin, Germany) that have a crystalline core of magnetite (Fe_3_O_4_) coated with Poly(diallyldimethylammonium chloride), with an average hydrodynamic diameter of 100 nm, a concentration of 25 mg Fe/mL, a particle number of ~1.8 × 10^15^/g, and a density of 1.25 g/cm^3^ with the R^4^N^+^ functional group.

#### 2.1.2. Evaluation of ION Size Polydispersity

ION’s size polydispersity was analyzed utilizing the Zetasizer Ultra system (Malvern, Worcestershire, UK) and the dynamic light scattering (DLS) technique. The analysis was carried out in a polypropylene cuvette containing IONs at a concentration of 50 µg Fe/mL, with the following equipment parameters: an angle of 173°, and an acquisition time of 5 s in a fixed position at 37 °C, with a thermal equilibrium period of 60 s.

#### 2.1.3. Evaluation of the Colloidal Stability of ION

The colloidal stability of ION in aqueous media was evaluated using the DLS technique, with the same configuration used in Section 2.1.2, and tested under the following conditions: (i) ION + water; (ii) ION + Dulbecco’s Modified Eagle’s Medium/Nutrient Mixture F12 (DMEM F12); (iii) ION + DMEM F12 + 10% fetal bovine serum (FBS); and (iv) ION + DMEM F12 + 20% FBS.

#### 2.1.4. Evaluation of the Zeta Potential of ION

The surface charge of IONs was measured through the zeta potential using the Zetasizer Ultra system (Malvern, Worcester, UK) at a concentration of 50 µg Fe/mL of ION in water, with pH adjusted to 7.6 at 37 °C. For this analysis, a small-volume polystyrene cuvette (DTS1070) was used.

#### 2.1.5. Evaluation of the Heating Potential of IONs for MHT Therapy

For evaluation of the heating potential of ION when exposed to the AMF of the MHT equipment, different combinations between of intensity between 50 and 300 Gauss and oscillation at frequencies of 305, 557, 715, and 874 kHz were tested. This MHT system comprised the DM100 applicator (nB nanoScale Biomagnetics, Zaragoza, Spain) and the DMC1 controller module (nB nanoScale Biomagnetics, Zaragoza, Spain), enabling therapeutic planning and execution. Fiber optic sensors were used for temperature monitoring in both in vitro and in vivo studies (Luxtron 3204, Lux-Tron Corporation, Northwestern Parkway, CA, USA).

#### 2.1.6. Specific Absorption Rate and Intrinsic Loss Power Calculation

Specific Absorption Rate (SAR) and intrinsic loss power (ILP) were calculated from the heating curves using the software Zar v1.0 (nB nanoScale Biomagnetics, Zaragoza, Spain). For the calculations, the relationship $SAR\;(W/g)=\frac{m_{ION}c_{ION}+m_{l}c_{l}\;}{m_{ION}}\;\left(\frac{dT}{dt}\right)\;max$ was used, where *m_ION_*: mass of the ION [kg]; *c_ION_*: specific heat of the ION [J/(kg.K)]; *m_l_*: mass of the liquid [kg]; *c_l_*: specific heat of the liquid [J/(kg.K)]; and (dT/dt) max: maximum gradient of the colloidal sample heating curve. Another way to evaluate the efficiency of heating mediated by ION was to use the ILP parameter using the formula ILP = SAR/(fx H_0_^2^), where f represents the frequency of oscillations in the magnetic field in kHz and H_0_ represents the intensity of the alternating magnetic field. ILP was used to compare heating efficiency when IONs were subjected to different alternating magnetic fields.

The appropriate combination of ION magnetic properties, optimization of SAR, and adjustment of magnetic field parameters are fundamental to the success of MHT therapy.

### 2.2. In-Vitro Study

#### 2.2.1. MDA-MB-231 Cell Culture

The study used the MDA-MB-231 cells, using the TNBC cell line from Banco de Células do Rio de Janeiro (BCRJ). These cells were genetically modified to adequately express the luciferase gene resistant to neomycin (MDA-MB-231/Luc) and cultured in DMEM F-12 medium, supplemented with 10% FBS (GIBCO^®^ Invitrogen Corporation, Carlsbad, CA, USA), and 1% antibiotic–antimycotic solution (GIBCO^®^ Invitrogen Corporation, Carlsbad, CA, USA), and were incubated in a humidified atmosphere with 5% CO_2_ at 37 °C (Thermo Fisher Scientific Inc. 3110, Waltham, MA, USA) until achieving the desired density of 90% confluence. For the trypsinization process, the trypsin EDTA was used (GIBCO Invitrogen Corporation, Carlsbad, CA, EUA).

#### 2.2.2. Evaluation of Kinetics of Bioluminescent Signal of MDA-MB-231 Cells

The peak timing of the bioluminescence imaging (BLI) signal, as well as the duration of the BLI signal, were determined by analyzing the BLI kinetics of MDA-MB-231 cells. A 96-well plate was used with a concentration of 1 × 10^6^ cells/well, including a control group without luciferase and an experimental group with the addition of 10 µL of D-Luciferin (30 mg/mL). The images were acquired by the IVIS^®^ Lumina LT Series III equipment (Perkin Elmer, Santa Clara, CA, USA), using signal intensity with automatic exposure time, an f/stop of 4, and a binning of 4, every 5 min over 8 h. The areas of interest were delineated with an area of 2 cm^2^ in each well, and signal processing (photons/s) took place using the Living Image version software IVIS Imaging System (Version 7.4.2) (Perkin Elmer, Santa Clara, CA, USA).

#### 2.2.3. Evaluation of Labeling of MDA-MB-231 Cells with ION and Their Internalization into Cells

The MDA-MB-231 cell labeling was carried out with five ION concentrations (10, 20, 30, 40, and 50 µg Fe/mL) dispersed in DMEM F12 for 18 h. Then, the cells of the wells were washed with phosphate-buffered saline (PBS) and fixed with 4% paraformaldehyde (PFA, Sigma-Aldrich, EUA). 

After cell labeling and fixed, ION internalization into cells was evaluated by blue-stained Prussian, using a 300 µL solution of 0.25 mg potassium ferrocyanide (Sigma–Aldrich, Saint Louis, MO, USA) and 5% hydrochloric acid (Merck, Darmstadt, Germany) in each well for 7 min, followed by PBS washing for image acquisition in a bright field to demonstrate the internalization of ION in MDA-MB-231 (in blue) using the Nikon TiE microscope (Nikon, Tokyo, Japan), with magnifications of 10× and 40×.

#### 2.2.4. Evaluation of Nanoparticle Toxicity for MDA-MB-231

ION internalization into the cells was evaluated the cytotoxicity exhibited by BLI under the same cell labeling conditions described in Section 2.2.3 and using the same parameters of BLI acquisition described in Section 2.2.2. The cell viability quantification was demonstrated using the relation between the BLI signal intensity of MDA-MB-231 cells labeled with ION in different ION concentrations (10, 20, 30, 40 e 50 µg Fe/mL) and unlabeled MDA-MB-231, multiplied by 100.

#### 2.2.5. Evaluation of Magneto-Hyperthermia Therapy in MDA-MB-231 Cells

In vitro MHT therapy evaluation was carried out using 1 × 10^6^ cells dispersed in 200 µL microtubes in three groups: (i) MDA-MB-231 + CMA, (ii) MDA-MB-231 + ION, and (iii) MDA-MB-231 + ION + CMA. A total of 20 µL of ION was added to the ION groups, and the MHT therapy was performed for 40 min at field parameters of 300 Gauss and an oscillation frequency of 420 kHz. The efficacy of MTH was evaluated to compare efficacy before and after AMF application in triplicate samples by BLI, acquired with the same parameters described in Section 2.2.2, after adding 10 μL of D-Luciferin to each sample. The BLI intensity quantification value was the maximum peak of the BLI signal. Quantification was expressed as the percentage of BLI intensity before and after the MHT process, and Living Image software was used to analyze the BLI signal, with a region of interest (ROI) of 1.8 cm^2^.

### 2.3. In-Vivo Study

#### 2.3.1. Animals

The study was designed and developed following the guidelines of the Ethics Committee on the Use of Animals (CEUA) of the Hospital Israelita Albert Einstein (HIAE). The project was approved by CEUA (number 4884-21). Isogenic female mice of the BALB/c Nude lineage, aged between 8 and 12 weeks and weighing between 17 and 25 g, were acclimated at the vivarium of the Centro de Experimentação e Treinamento em Cirurgia (CETEC) of the Instituto Israelita de Ensino e Pesquisa Albert Einstein (IIEPAE), accredited by the Association for Assessment and Accreditation of Laboratory Animal Care (AAALAC). These animals were exposed to 21 ± 2 °C with a 12 h light/dark cycle, and the access to food and water was ad libitum during the experiment.

#### 2.3.2. Experimental Design

The in vivo experimental design was divided into two stages: the tumor growth evaluation and the therapeutic process using MHT therapy with or without physical training and omega-3 supplementation.

First stage: Tumor growth was evaluated by BLI techniques (7, 14, 21, and 28 days after tumor induction) and histology (28 days after tumor induction) in three experimental groups (n = 8 in each group). After the tumor growth assessment period, the tumors were collected from the right flank region through surgical removal of the tissue, before being fixed in 10% formalin (Sigma-Aldrich, USA), and embedded in paraffin (Sigma-Aldrich, USA). Then, 3 µm histological sections were obtained and placed on silanized histological slides (Starfrost, Lowestoft, UK) for hematoxylin–eosin staining (HE, EasyPath, Indaiatuba, São Paulo, Brazil) and visualized using a DM750 optical microscope (Leica, Wetzlar, Germany).

Second stage: The efficacy of different therapeutic combinations was evaluated by BLI imaging in eight experimental groups (8 animals per group), as shown in Table 1. Experimentation lasted for 21 days after tumor induction, and animals were sacrificed at the end of this period.

**Figure 1:**
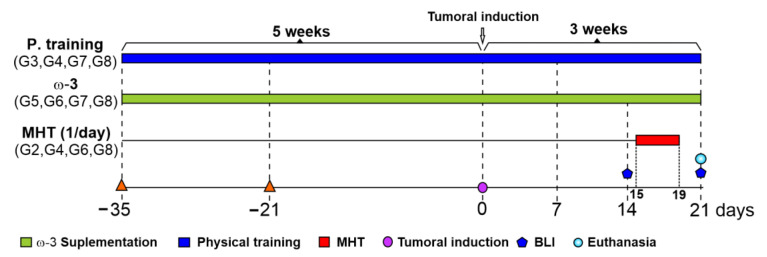
The timeline of the in vivo experimental design of the therapeutic approaches, physical training, omega-3 supplementation, and magneto-hyperthermia therapy. Abbreviations: MHT: magneto-hyperthermia; ω-3: omega-3; BLI: bioluminescence imaging. Note: G1 (control); G2 (only MHT); G3 (only physical training); G4 (physical training + MHT); G5 (only omega-3); G6 (omega-3 + MHT); G7 (physical training + omega-3); G8 (physical training + omega-3 + MHT).

The in vivo experimental design schematize is depicted in Figure 1. The G3, G4, G7, and G8 groups underwent physical training for five weeks before tumor induction and three weeks after; the G5, G6, G7, and G8 groups were supplemented with omega-3 during the eight weeks; the G2, G4, G6, and G8 groups were treated with MHT therapy for five consecutive days on the 8th week, starting from 15 to 19 days after tumor induction. BLI images were used to evaluate the therapeutic process between 14 and 21 days following tumor induction.

#### 2.3.3. Omega-3 Supplementation

Over eight weeks (5 weeks before and 3 weeks after tumor induction), the animals received omega-3 supplementation via daily gavage, comprising approximately 300 µL of fish oil, Super Omega-3TG (Essential Nutrition, São José, Santa Catarina, Brazil). This fish oil contained 360 mg of eicosapentaenoic acid (EPA) + 240 mg of docosahexaenoic acid (DHA) per mL, at a dose of 5.4 g/kg of EPA and 3.6 g/kg of DHA.

#### 2.3.4. Physical Training

Before starting the experimental program, the animals were screened for three consecutive days to ensure a good running performance on the monitored treadmill for rodents (AVSProjetos, São Paulo, SP, Brazil) using the Dischman behavioral scale [44]. The animals with good scores were selected for the exercise groups. The next day, we evaluated the maximum speed of animals using the treadmill equipment to determine the eight-week progressive physical training program (five weeks before and three weeks after tumor induction).

Daily sessions lasted 30 min, with a percentage progression of weekly speed based on the individual maximum speed of each initial animal, ranging from 40% to 80% and increasing by 10% per week; in the last three weeks, this was maintained 80%. This method ensured continuous stimulation of the animal’s performance, avoiding exhaustion. The Dischman score was used throughout training to track the animal’s progress and identify indications of weariness.

#### 2.3.5. Magneto-Hyperthermia Therapy

After 15 days of tumor induction, the MHT therapy started. Firstly, 20 µL of ION was administrated in two equidistant points from the center of the tumor using the 10 µL Hamilton syringe (Hamilton Company, Reno, NV, USA). After 4 h of ION distribution into the tumor, the animals were maintained under isoflurane inhalation anesthesia (2% saturation) and positioned inside the coil of the MHT equipment. The MHT procedure lasted 40 min and the animal was subjected to a controlled heating regime in two distinct stages. In the first stage, a 300 Gauss magnetic field was applied, oscillating at a frequency of 420 kHz until reaching a temperature of 42 °C (considered therapeutic), and in the second stage, the frequency remained constant and the intensity of the magnetic field was modulated to maintain the temperature of 42 °C. To monitor temperature, a 550 µm optical fiber (Luxtron Corporation, Northwestern Parkway, CA, USA) was inserted into the tumor during the procedure. 

MHT was repeated for three consecutive days. On the fourth day, another 20 µL of ION was added in the same way to ensure a similar amount of ION was added to the tumor, and the therapeutic process was repeated for two more consecutive days, totaling five applications of the AMF and concluding the therapeutic process on the 19th day after tumor induction.

### 2.4. Statistic Analyses

Data were presented as a mean and standard deviation; their normality was assessed using the Shapiro–Wilk test. For the normal data, the group comparisons were assessed using Student’s *t*-test or ANOVA, and for non-normal data, the Mann–Whitney test or Kruskal–Wallis test were used. A post hoc analysis was conducted to compare more than three significant groups with Bonferroni correction for multiple comparisons. The significance level was *p* < 0.05, and the analyses were carried out using JASP 0.17.3 software. (http://www.jasp-stats.org; accessed on 10 May 2023) and Origin 2023 (OriginLab, Northampton, MA, USA).

## 3. Results

### 3.1. ION Physicochemical Evaluation

**Figure 2:**
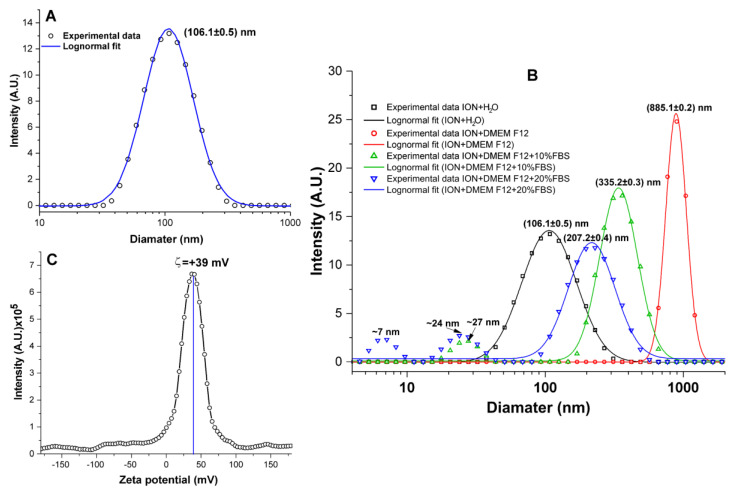
ION physicochemical evaluation. (**A**) ION polydispersity curve fitted to a log-normal curve; the circles (O) represent the experimental data and the solid line (-) represents the fit to the Log-normal curve. (**B**) The curve of intensity (U.A.) versus diameter (nm), showing the polydispersity of the hydrodynamic diameter of the ION and indicating the values of the maximum peaks for the samples under the following conditions: (i) ION + water in black curve; (ii) ION + DMEM F12, in red curve; (iii) ION + DMEM F12 + 10% FBS in green curve; and (iv) ION + DMEM F12 + 20% FBS in blue curve. (**C**) Distribution of the zeta potential of IONs with a magnetite core covered with Poly (diallyldimethylammonium chloride) dispersed in water, and at pH 7.4, indicating the maximum peak at +39 mV.

The evaluation of the ION physicochemical characteristics showed an average hydrodynamic diameter of 106.1 ± 0.5 nm, compatible with the value provided by the manufacturer (100 nm), as shown in Figure 2A. An adequate colloidal ION stability was shown when tested in biological medium (DMEM F12) supplemented with 10% or 20% of FBS (green curve of Figure 2B), where their diameter was maintained at 335.2 ± 0.3 and 217.2 ± 0.4 nm, respectively. In the medium without FBS, the diameter increased to 885.1 ± 0.2 nm (red curve of Figure 2B), showing the formation of ION agglomerations that can interfere with ION’s internalization into cells. Another important ION characteristic was a peak zeta potential around +39 mV (Figure 2C); the positive ION surface charge favors a good interaction with the negative surface charge of cells.

### 3.2. Evaluation of the Heating Potential of IONs for MHT Therapy

The evaluation of ION’s heating potential for MHT therapy revealed that, in all tested AMF parameter combinations, the heating rate rose directly with increases in AMF frequency and intensity, achieving the goal temperature in less time. To reach the therapeutic temperature of 42 °C, we found that, at the frequency of 309 kHz and 200 Gauss, it took 65 s, and with 200 Gauss, the time required was 31.6 s. At a frequency of 364 kHz, the time required was 45 s combined with 300 Gauss and 23.6 s with 200 Gauss; at the frequency of 420 kHz, the time was 36 s (200 Gauss) and 18.6 s (300 Gauss); at the highest tested frequency (557 kHz), we observed the shortest times of 24.2 s (200 Gauss) and 13 s (300 Gauss). Accordingly, the time needed to achieve the therapeutic temperature was inversely proportional to the oscillation frequency and magnetic field intensity. 

**Figure 3:**
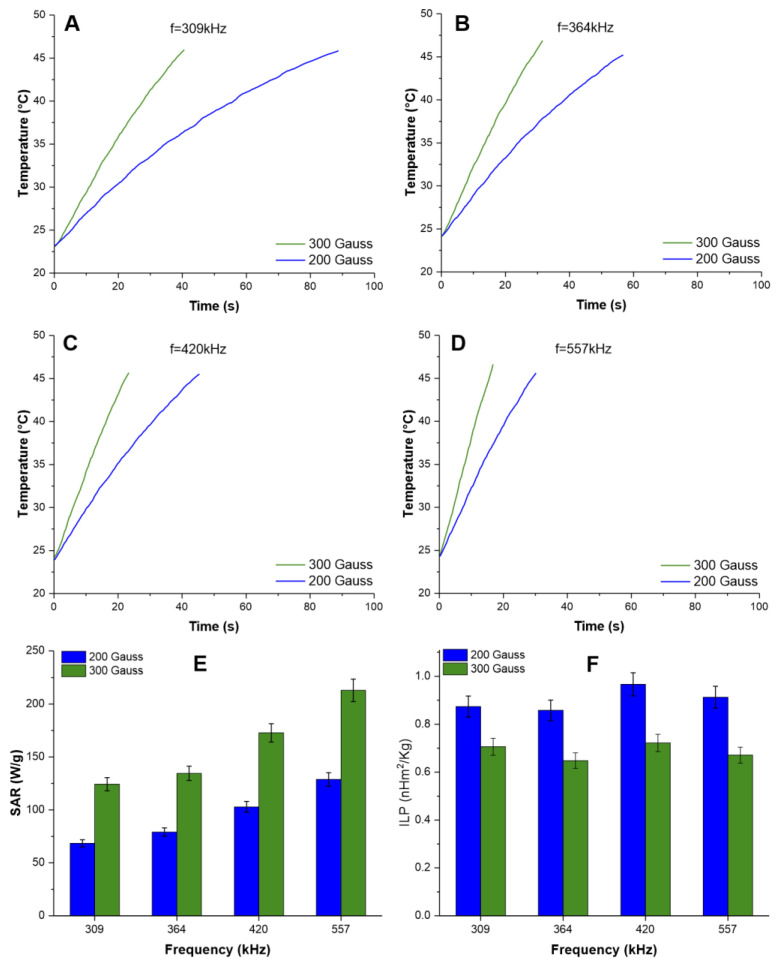
Heating curves of ION dispersed in an aqueous medium subjected to different conditions of alternating magnetic field oscillation frequency and magnetic field intensity: (**A**) 309 kHz; (**B**) 364 kHz; (**C**) 420 kHz; and (**D**) 557 kHz. The graphs show the results for a magnetic field of 200 Gauss (green line) and 300 Gauss (blue line). (**E**) SAR values as a function of the magnetic field intensity (200 and 300 Gauss) combined with the magnetic field oscillation frequencies (309, 364, 420, and 557 kHz). (**F**) ILP intrinsic power loss as a function of the AMF oscillation frequency, characterized by frequencies of 309, 364, 420, and 557 kHz, using magnetic fields of 200 (blue bars) and 300 Gauss (green bars).

From these heating curves (Figure 3A–D), the SAR and ILP values of the IONs were calculated, as shown in Figure 3E,F, respectively. The highest SAR value was obtained at a frequency of 557 kHz and 300 Gauss (213.0 ± 10.6 W/g, green bar), compared to other field strength and frequency conditions. The SAR values for 200 Gauss were 68.4 ± 3.4 W/g (309 kHz), 79.0 ± 4.0 W/g (364 kHz), 102.9 ± 5.1 W/g (420 kHz), and 128. 7 ± 6.4 W/g (557 kHz), and for 300 Gauss the SAR values were 124.3 ± 6.2 W/g (309 kHz), 134.5 ± 6.7 W/g (364 kHz), 172.8 ± 8.6 W/g (420 kHz), and 213.0 ± 10.6 W/g (557 kHz) (Figure 3E). 

However, the ILP values of the IONs subjected to heating with 200 and 300 Gauss and at all measured magnetic field oscillation frequencies (309, 364, 420, and 557 kHz) did not vary significantly, presenting an average of 0.903 ± 0.049 nHm^2^/kg for 200 Gauss and 0.819 ± 0.061 nHm^2^/k for 300 Gauss, without being influenced by magnetic field or frequency (Figure 3F).

### 3.3. In Vitro Study

#### 3.3.1. Evaluation of Kinetics of BLI of MDA-MB-231, and, after Labeling with ION, Their Internalization, and Cytotoxicity

**Figure 4:**
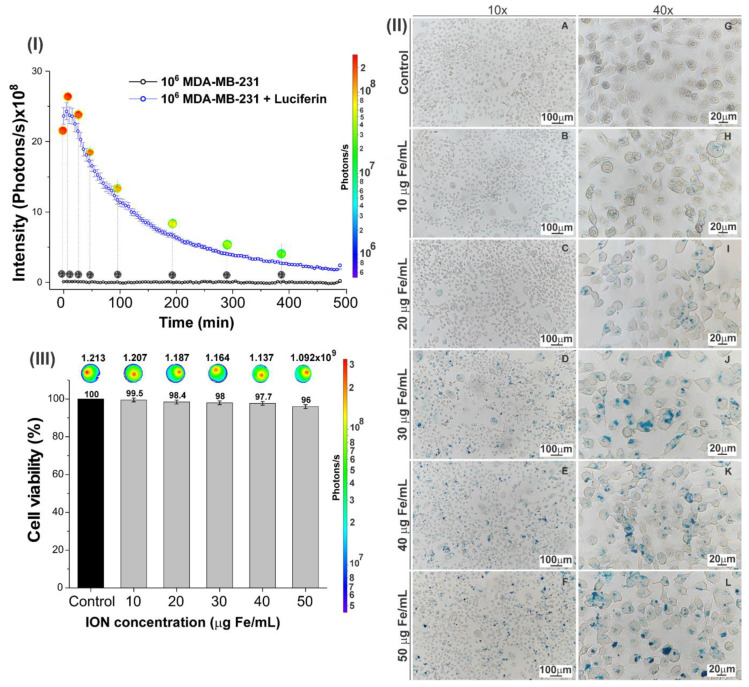
(**I**) BLI kinetics of MDA-MB-231 cells at a concentration of 1 × 10^6^ for both the control group (without the addition of luciferin) and the group with the addition of luciferin, considering the BLI decay signal until 480 min. At some points on the curve, the BLI image shows variations in intensity according to the scale bar. (**II**) Visualization by Prussian blue staining of the internalization of ION in MDA-MB-231 cells evaluated at concentrations of 0 (AG), 10 (BH), 20 (CI), 30 (DJ), 40 (EK), and 50 (FL) µg Fe/mL ION after cell labeling in 10× and 40×. (**III**) Relative viability (%) after labeling MDA-MB-231 with ION (overnight) at concentrations of 10, 20, 30, 40, and 50 µg Fe/mL using the BLI technique. Above each bar, the BLI intensity correspondence is shown.

The kinetics of the BLI signal of MDA-MB-234 cells were evaluated under two conditions, with and without the addition of luciferin, for the control conditions (blue and black curves of Figure 4I). The maximum BLI signal intensity of the cell from the kinetics curve was 24.4 × 10^8^ photons/s at 9.4 s, then the signal decayed until 480 min of measurement due to the adenosine triphosphate (ATP) consumed by transfected cells.

After the ION labeling process in these transfected cells was evaluated, the process of internalization began with different concentrations in IONs using Prussian blue staining, in which the ION concentration is highlighted by a blue color in cells. It was evident that with the increase in the ION concentration in the labeling, their internalization in the cells also increased, as shown in Figure 4II(A–L).

After the labeling process, we evaluated the cytotoxicity using the BLI technique, which showed a slight decrease in BLI signal intensity with the increase in ION concentration in the labeling process: 1.213 × 10^9^ (control); 1.207 × 10^9^ (10 µg Fe/mL); 1.187 × 10^9^ (20 µg Fe/mL); 1.164 × 10^9^ (30 µg Fe/mL); 1.137 × 10^9^ (40 µg Fe/mL); and 1.092 × 10^9^ photons/s (50 µg Fe/mL). From these BLI intensities, the percentage of cell viability was calculated, as shown in Figure 4III: 99.5% for 10 µg Fe/mL, 98.4% for 20 µg Fe/mL, 98.0% for 30 µg Fe/mL, 97.7% for 40 µg Fe/mL, and 96% for 50 µg Fe/mL. Thus, cell viability was shown to be dependent on the applied dose, with a toxicity of 4% for the maximum concentration that was evaluated.

#### 3.3.2. Evaluation of Efficacy of Therapeutic Magneto-Hyperthermia 

**Figure 5:**
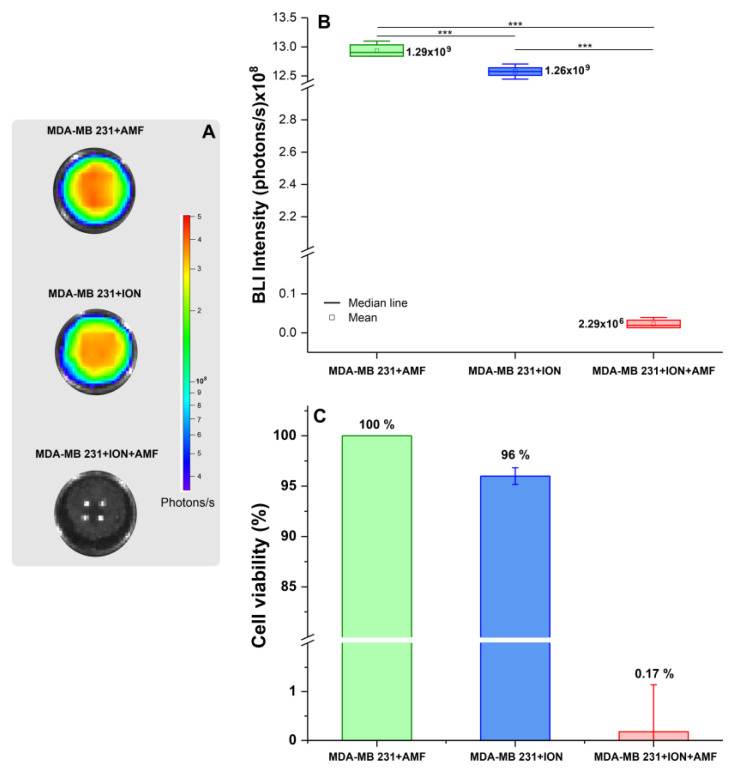
Assessment of the efficiency of in vitro MHT in experimental groups according to BLI signal intensity. (**A**) BLI of the three experimental conditions (MDA-MB-231 cells + AMF, MDA-MB-231 cells + ION, and MDA-MB-231 cells + ION + AMF); (**B**) boxplot of BLI intensities of the three experimental groups; (**C**) histogram showing the efficacy percentage of in vitro MHT therapy in the experimental groups according to BLI signal changes. ***: *p* < 0.001.

The decrease in the BLI signal indicates the effectiveness of the MHT technique in vitro, as it is directly related to cell viability (Figure 5A). The BLI signal in the MDA-MB-231 + AMF group was (1.294 ± 0.013) × 10^9^ photons/s, similar to that in the MDA-MB-231 + ION group with (1.258 ± 0.011) × 10^9^ photons/s. In the MDA-MB-231 + ION + AMF group, in which AMF was applied in the presence of ION, there was a marked decrease in the BLI signal concerning the other groups with (2.293 ± 0.012) × 10^6^ photons/s, as shown in Figure 5B. The ANOVA test showed a significant difference between the groups (*p* < 0.001), with a significant difference corrected by Bonferroni between the groups being evident in the post hoc analysis (*p* ≤ 0.001), as shown in Table 2. The decrease in the BLI signal was 4% in the presence of ION and 99.83% in the presence of ION and AMF (Figure 5C).

### 3.4. In Vivo Study

#### 3.4.1. Evaluation of the Tumor Growth by BLI and Histology

**Figure 6:**
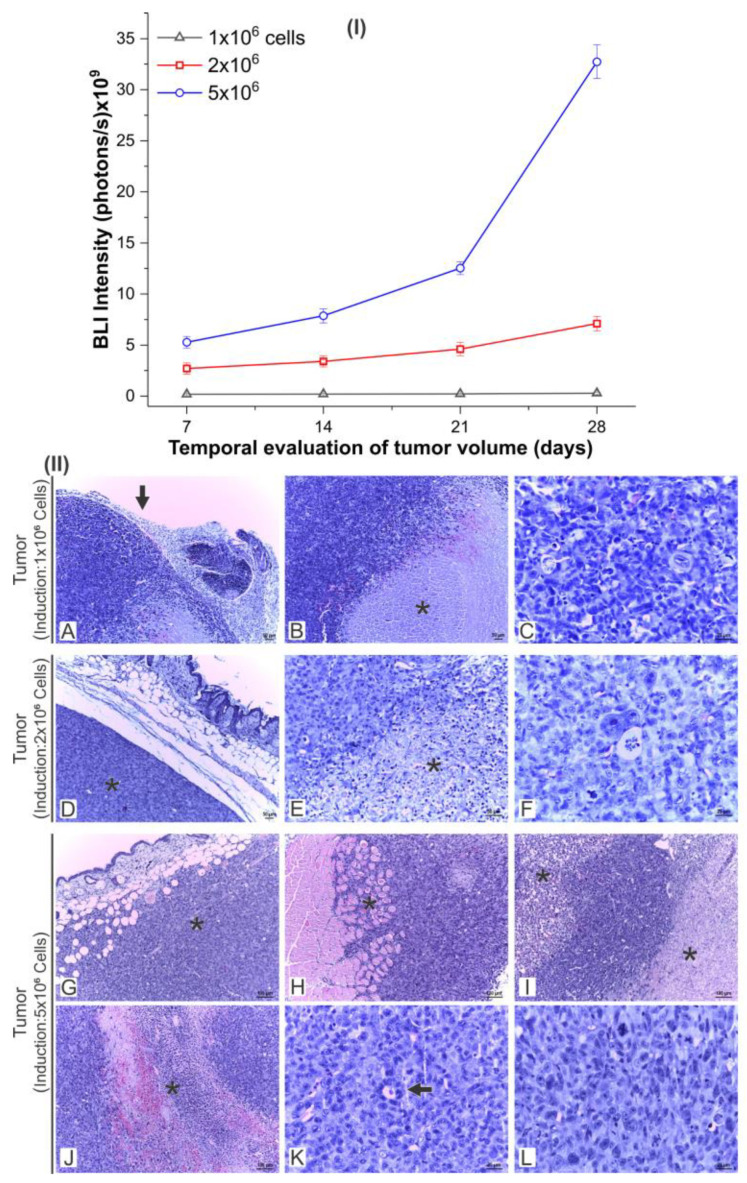
Evaluation of longitudinal tumor growth (**I**) according to BLI signal intensity in tumor tissue at 7, 14, 21, and 28 days after tumor induction in three experimental groups induced with 1 × 10^6^, 2 × 10^6^, and 5 × 10^6^ MDA-MB-231 cells, and (**II**) according to histological sections after 28 days of tumor induction. The tumors were induced with 1 × 10^6^ cells (**A**–**C**); 2 × 10^6^ cells (**D**–**F**) and 5 × 10^6^ cells (**G**–**L**). (A-arrow) Neoplastic epithelial neoformation, hypercellular, and encapsulated; (B*) neoplasm and marked pleomorphism, adjacent to a large necrotic site; (**C**) neoplastic epithelial population exhibiting marked pleomorphism and numerous mitoses; (D*) nodular neoplastic in the subcutaneous layer; (E*) neoplasm, marked pleomorphism, and large necrotic site; (**F**) neoplasm, pleomorphic tumor cells, and numerous mitotic figures; (G*) nodular and hypercellular neoformation in the subcutaneous layer and fatty infiltration; (H*) infiltrative neoplastic cell population in the muscular layer; (I*) neoplasm and marked pleomorphism, marginalized by large necrotic sites; (J*) extensive necro-hemorrhagic site; (K-arrow). Formations were made in a tubular arrangement: (**L**) neoplasm, marked cellular pleomorphism, and numerous mitoses.

A longitudinal evaluation of tumor growth with BLI (7, 14, 21, and 28 days after tumor induction) was performed with the three cell concentrations used in the tumoral induction (1 × 10^6^, 2 × 10^6^, and 5 × 10^6^ cells) to determine the best conditions for the application of MHT therapy. The intensity of the BLI signal in the tumor volume of animals that were induced with 1 × 10^6^ cells varied from 1.85 × 10^9^ photons/s (7th day) to 2.84 × 10^9^ photons/s (28th day), increasing by approximately 54% (gray triangle/line of Figure 6I). When induced with 2 × 10^6^ cells, the tumor growth was 163%, with the BLI intensity ranging from 2.7 × 10^9^ photons/s (7th day) to 7.1 × 10^9^ photons/s (28th day) (red square/line of Figure 6I). When an induction with 5 × 10^6^ cells was used, the tumor increased by 517% between the 7th (5.3 × 10^9^ photons/s) and 28th day (3.3 × 10^10^ photons/s; blue circle/line of Figure 6I).

Tumor growth assessed by histological sections after 28 days of tumor induction by 1 × 10^6^, 2 × 10^6^, and 5 × 10^6^ cells revealed a predominance of similar tissue characteristics in tumor lesions, induced by different cell concentrations, at different intensities according to the cell concentration that was used. The animals subjected to the two lowest cell concentrations exhibited the same lesional pattern (1 × 10^6^ and 2 × 10^6^), characterized by hypercellular neoplastic neoformation, circumscribed and encapsulated in the subcutaneous tissue, composed of a population of epithelial cells of marked pleomorphism, moderate necrotic sites, and numerous mitotic figures (Figure 6II(A–L)). However, the neoformation induced by the higher concentration of cells (5 × 10^6^) exhibited worsening of the malignancy criteria, such as the loss of subcutaneous delimitation and restriction, presenting areas of infiltration of the subcutaneous and muscular layers. Due to the exacerbated growth, the necrotic and hemorrhagic areas were more extensive and pronounced (Figure 6II(G–I)). Histopathological findings suggest that cellular concentrations ≥5 × 10^6^ are favorable to exacerbated tumor growth, causing local infiltration and worsening of intratumoral necrosis. Therefore, considering the longitudinal monitoring of the tumor, the 2 × 10^6^ concentration demonstrated the ideal tumor development within the necessary limits for an evaluation of the effectiveness of the magneto-hyperthermia technique. 

Considering the evaluation of the animals’ clinical signs, BLI, and histological evaluation, it was suggested that therapy be started on the 14th day after tumor induction with 2 × 10^6^ cells.

#### 3.4.2. Evaluation of the Efficacy of Therapeutic Combinations: Omega-3 Supplementation, Physical Training, and Magneto-Hyperthermia

**Figure 7:**
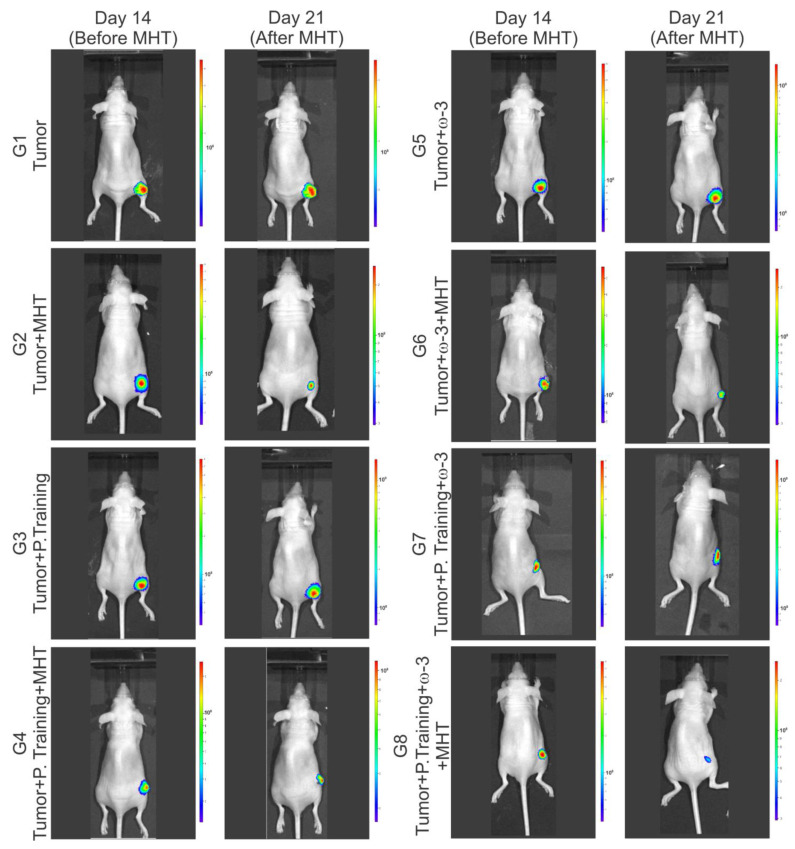
Assessment of the effectiveness of therapies regarding tumor growth via imaging and bioluminescence signal intensity. Bioluminescence imaging (BLI) of the experimental groups before (14 days after tumor induction) and after magneto hyperthermia (MHT) therapy (21 days after tumor induction).

The evaluation of the efficacy of MHT therapy was carried out using the BLI technique, comparing the average BLI signal of the tumors on the 14th day (before MHT) and the 21st day (after MHT) after tumor induction in each experimental group: G1 (control), G2 (only MHT), G3 (only physical training), G4 (physical training + MHT), G5 (only omega-3), G6 (omega-3 + MHT), G7 (physical training + omega-3), and G8 (physical training + omega-3 + MHT), as shown in Figure 7, with a clear decrease in signal in the pre- compared to post-MHT images in some groups (G2, G4, G6, and G8). 

**Figure 8:**
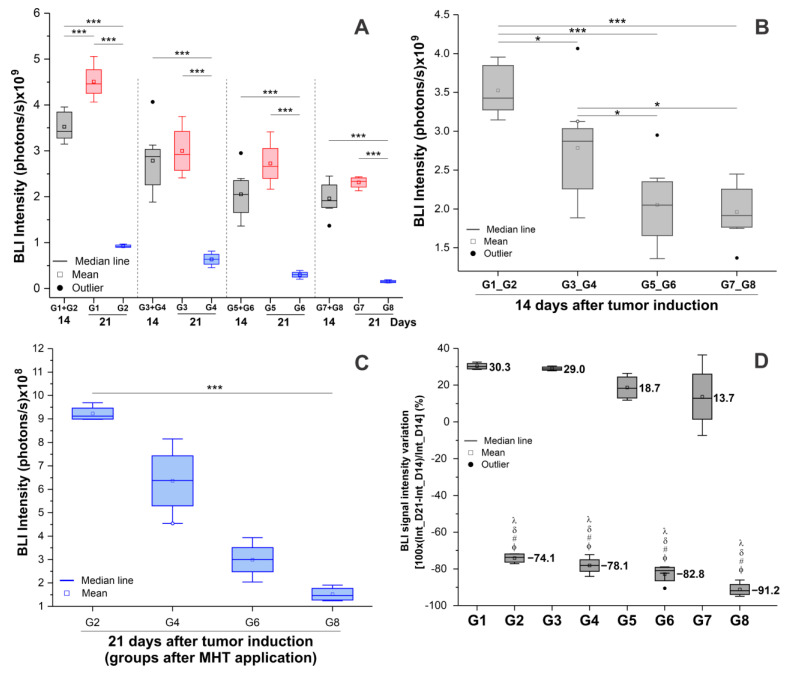
Assessment of the effectiveness of therapies regarding tumor growth via bioluminescence (BLI) signal intensity. (**A**) Assessment of BLI intensities in the experimental groups before and after the application of MHT therapy: the pre-groups in gray with the same condition are shown in gray; the post-groups that did not receive MHT therapy are shown in red; the groups that received the MHT are shown in blue. (**B**) Analysis of the groups in the pre-MHT therapy condition, with a difference observed in the BLI signal when evaluating the groups that underwent 14 days of other adjuvant therapies. (**C**) Analysis of only the groups that received MHT therapy and their evaluation regarding the BLI sign. (**D**) Boxplot of the effectiveness (%) of the MHT therapeutic process between the experimental groups. Note: G1 (control); G2 (only MHT); G3 (only physical training); G4 (physical training + MHT); G5 (only omega-3); G6 (omega-3 + MHT); G7 (physical training + omega-3); G8 (physical training + omega-3 + MHT); G1 + G2 (control); G3 + G4 (only physical training); G5 + G6 (only omega-3); G7 + G8 (physical training + omega-3; *: *p* < 0.01; ***: *p* < 0.001. ϕ—significant difference (*p* < 0.001) when compared with G1; #—significant difference (*p* < 0.001) when compared to G3; δ—significant difference (*p* < 0.001) when compared to G5; λ—significant difference (*p* < 0.001) when compared with G7.

For BLI signal quantification, we grouped the groups with the same initial conditions—G1 plus G2 (controls), G3 plus G4 (only physical training), G5 plus G6 (only omega-3), and G7 plus G8 (physical training + omega-3)—to compare each condition after MHT (G2, G4, G6, and G8), as shown in the graph in Figure 8A. 

In the longitudinal analysis intragroup of groups without MTH (G1, G3, G5, and G7), the tumor growth was only a significant (*p* < 0.001) measurement when considering BLI signal in the control group (G1 + G2 versus G1), whereas, when evaluating the efficacy of the group that applied the MHT therapy alone (G2) or associated with other therapies (G4, G6, and G8), the BLI signal decreased significantly (*p* < 0.001) in all conditions (G3 + G4 versus G4, G5 + G6 versus G6 and G7 + G8 versus G8; gray and blue boxplots of Figure 7A and Table 3).

In the transversal evaluation between groups (G1 plus G2, G3 plus G4, G5 plus G6, and G7 plus G8), only pre-MHT (14th day after tumor induction; Figure 8B) showed a significant difference between groups according to the ANOVA test (*p* < 0.001), and the post hoc test (Table 4) showed that the mean BLI signal in the control group (G1+ G2; 3.524 × 10^9^ ± 3.155 × 10^8^ photons/s) was significantly higher than in the groups that received physical training (G3 + G4; 2.785 × 10^9^ ± 6.769 × 10^8^ photons/s; *p* = 0.03), omega-3 (G5 + G6; 2.055 × 10^9^± 5.100 × 10^8^ photons/s; *p* < 0.001), or both treatments (G7 + G8; 1.961 × 10^9^ ± 3.495 × 10^8^ photons/s; *p* < 0.001). The combined therapy showed the lowest average BLI signal compared to the other groups, and compared to other therapies, the results were only significantly lower than the physical training group (G3 + G4; *p* = 0.012). Interestingly, the omega-3 supplementation showed significantly more effectiveness than physical training (*p* = 0.033), as shown in Table 4 and Figure 8B, and similar results were obtained when compared with the combination of physical training and omega-3 (G7 + G8; *p* = 1.000).

In the transversal analysis, only conditions after 21 days with and without the MHT procedure (G1 × G2, G3 × G4, G5 × G6, and G7 × G8) showed a significant (*p* < 0.001) reduction in BLI signal in the groups that received MHT, as shown in Table 5 and Figure 8A (red and blue boxplots). Therefore, the treatment with MHT was more effective than the adjuvant treatment, either alone or combined with exercise and omega-3 supplementation.

Furthermore, we only analyzed the BLI signal in the groups that received MHT treatment (G2, G4, G6, and G8), as shown in Figure 8C. It is evident that when we examine any of the adjuvant therapies (physical exercise, as in G4m or omega-3, as in G6), the reduction is greater than the control (only MTH), but the result was only significant (*p* < 0.001) when both adjuvant therapies (physical exercise and omega-3, G8) were compared to the control (G2), as shown in Table 6. Therefore, the adjuvant therapies have the effect of delaying tumor growth (Figure 8B,D) and help to reduce the BLI signal when associated with MTH (Figure 8C,D), but they were more effective than the control when both were administered together, as opposed to when used in an isolated form.

Analyzing all experimental groups regarding the percentage effectiveness of the BLI signal, we can observe that, in all groups that were not subjected to MHT therapy, the signal had a positive percentage increase (G1, G3, G5, and G7). This was greater in G1, which did not receive any adjuvant treatment, showing a BLI signal increase of 30.3% over time (from the 14th day to the 21st day), as shown in Figure 8D and Table 7. The groups that received MHT therapy (G2, G4, G6, and G8) all show a percentage decrease in efficacy, representing a reduction in the BLI signal when comparing BLI pre- and post-MHT treatment, with greater efficacy in G8, which showed a 91.2% reduction in BLI signal (Table 7 and Figure 8C,D).

## 4. Discussion

This study showed that, for the triple-negative breast cancer model, adjuvant therapies such as omega-3 supplementation and physical training delay tumor growth, and when combined and associated with magneto-hyperthermia therapy, their efficiency in tumor reduction, as analyzed by BLI signal intensity, was much more effective. Accordingly, MTH associated with exercise and omega-3 is a promising treatment for this type of cancer, which is considered the most aggressive due to its rapid growth, evidence of early metastasis, greater probability of recurrence after treatment, and low survival [45].

BC represents a growing global concern, especially for the female population of working age. Its pathophysiology presents a series of challenges, including the need to identify new prevention and treatment strategies, aiming to improve patients’ quality of life affected by this disease [46,47,48,49]. Recent studies have provided promising evidence of the benefits of omega-3 supplementation and complementary therapies in combination with chemotherapy, radiotherapy, and surgical procedures for patients with BC [50,51,52,53].

A recent systematic review developed by our group showed that supplementation with omega-3 has an effect on prevention and treatment in different models of breast cancer, as well as being considered a good adjuvant in conventional treatments, such as chemotherapy, minimizing side effects and enhancing therapeutic action. This review also showed that tumor induction by cell transplantation (MDA-MB-231) was the most used model for BC therapeutic evaluations, associated mainly with the consumption of DHA and EPA, which presented a wide variation in doses and the form of administration [54]. Due to this lack of consensus on the ideal does to ensure the effective action of omega-3 in breast cancer, in this study, an EPA dose of 5.4 g/kg per animal and a DHA does of 3.6 g/kg per animal, administered by gavage, was established. This dose was within the range found in our review [54] and in the literature [55]. However, the dose of supplemental omega-3 PUFAs known to be beneficial for certain treatments or aging processes in humans is already well-established according to the global standard for EPA and DHA intake: for cardiovascular diseases, at least 500 mg/day of EPA + DHA is recommended; pregnant and lactating women, DHA of 300 mg/day is recommended, and for general adults, 300–400 mg EPA + DHA/day is recommended. A slight modification to the guideline was reported in Brazil for women with coronary artery disease (1 g/day EPA + DHA) and those who were pregnant or nursing (DHA-200 mg/day), and in France, 500 mg of EPA + DHA was advised daily to lower the risk of colon and breast cancer [56]. The administration of 2 g of DHA daily has been shown in a recent review study to exhibit safety and good absorption, and to saturate plasma levels based on human distribution, interconversion, and dosage response [57].

It is recognized that leading a healthy lifestyle both lowers the chance of acquiring breast cancer and improves the prognosis for the disease, and that regular exercise has positive impacts on cancer-related traits that can help slow the progression of the disease [58,59]. Physical training improves muscular strength, psychological well-being, and cardiorespiratory fitness. These can reduce the incidence of depression and anxiety, which are frequently linked to the side effects of chemotherapy [60,61]. Physical training can also alleviate serious adverse effects following the treatment of breast cancer [62]. On the other hand, opinions regarding the best and most appropriate type of training for breast cancer remain unclear (including the best modality, time, and duration, among other aspects) [63]. According to a systematic review, women who have survived breast cancer have been shown to benefit from moderate- to high-intensity exercise sessions, and among the various exercise modalities, resistance exercise demonstrated effects of as high as 55% for one-repetition maximum exercises, either alone or in conjunction with other training regimens like impact, high-intensity interval training (HIIT) or aerobics (48% of heart rate). Furthermore, the primary advantages were heightened physical strength, which can be achieved by resistance training, either alone or in conjunction with other forms of exercise; reduced fatigue; enhanced quality of life; enhanced psychological impacts; and more free time [64]. The effects of a 12-week physical training program in the preclinical trial with a triple-negative breast cancer model also included a smaller tumor mass, and the mitochondria in the tumors presented lower respiratory rates in the state of maximum electron transport capacity and a higher expression of genes related to tumor suppressors. Therefore, exercise thereby slowed the tumor’s growth, affecting the macronutrients and mitochondria metabolisms [65]. Furthermore, the clinical trials focusing on supplementation with daily high-dose vitamin D3 plus omega-3, combined with a simple home strength exercise program, showed a cumulative reduction in the cancer risk in generally healthy and active adults over 70 years [42].

MHT represents a promising alternative to tumor therapy in pre-clinical analysis, but for clinical treatment it is not yet a reality, and the number of pre-clinical studies that can be translated into clinical trials is minimal. This situation is sometimes aggravated by the lack of critical information about a particular aspect of the procedure, as pre-clinical studies have used very varied conditions for the application of MHT, such as different nano-formulations (size, coating, composition, shape, etc.) and different AMF parameters (oscitation frequency and magnetic field), resulting in distinct temperature ranges that can cause apoptosis (41 to 43 °C), or thermoablation necrosis (>45 °C), where heat-induced protein denaturation causes disruption of the cytoskeleton and membrane, altering the conformation of deoxyribonucleic acid (DNA), among other molecular effects [66]. MHT therapy has been applied very efficiently in the treatment of different types of tumors in pre-clinical studies [67].

The physicochemical characteristics of the nanoformulation used in this study were evaluated before MHT application to allow for the best therapeutic conditions to be used in vitro and in vivo. The ION distribution was adequate in water, but when dispersed in the culture medium (DMEM F12), they tended to agglomerate. With the addition of 10% FBS, the agglomeration was minimized, and with a higher concentration of FBS (20%), the agglomeration was even smaller (Figure 2B) due to the forces present that affected the stability of the suspended nanoparticles [68,69]. The forces that participate in the interaction between the of the ION electrical charges and the surrounding liquid medium are mainly electrostatic, steric repulsion, Van Der Waals, hydrodynamic, and gravitational forces, which can influence the agglomeration, attraction or dispersion of molecules, and sedimentation [70,71,72]. This may impair their internalization in tumor cells, and consequently the effectiveness of anti-tumor treatment using MHT therapy.

Another relevant aspect of the analysis of ION is its heating potential, which was assessed using SAR values (Figure 3E), to ensure that the MHT would be successful [73,74]. Therefore, the therapeutic planning that was undertaken to determine the time needed to reach the temperature of 42 °C was based on an in vitro experiment with the combination of 4 AMF oscillation frequencies (309, 364, 420, and 557 kHz) and two AMF intensities (from 200 to 300 Gauss). It was found that the increase in these parameters was inversely proportional to time, and the intensity of 300 Gauss and the frequency of 420 kHz were adopted for the in vivo experiment (Figure 3D), as they presented the highest SAR value of (213.0 ± 10.6) W/g. This was similar to studies found in the literature with other types of nanoparticles, which proved to be effective in MHT [75,76,77,78,79,80]. A high SAR value improves the efficiency of magnetic energy transfers and heat generation for cancer cells, influencing the effectiveness of MHT therapy [78,81,82,83,84]. 

When evaluating the interaction of IONs in MDA-MB-231 tumor cells, we found that the highest concentration of ION used (50 µg Fe/mL) in the cell labeling process led to greater internalization into cells, as observed by the conventional method of Prussian blue staining (Figure 4II), and a low impairment of cell viability (4%) was found by the BLI (Figure 4III). The BLI is useful when attempting to visualize and monitor cell physiology processes such as cell viability and proliferation [85], such as the tumor growth evaluation [86] used in this study (Figure 6I). The same nanoparticle was evaluated and labeled in A549 cells (a human lung carcinoma cell line), using a concentration that was twice as high as that used in our study (100 µg Fe/mL), and showed a reduction of just 6% in cell viability [87].

Regarding the BC model, we used a cell transplantation model for the in vivo study, which is thought to be advantageous due to its short cycles, low costs, minimal variation, and high rates of tumor growth when compared to genetically engineered mice and the induced models (biological, physical, and chemical) [88]. The challenge in this model was to determine the ideal tumor growth conditions, such as the amount of cells that were administered, the time of tumor growth, and the tumor size at the start of the therapeutic processes, respecting the animal’s quality of life during the experiment. In the longitudinal assessment of tumor growth by BLI (Figure 6), we observed that the highest cellular concentrations (2 × 10^6^ and 5 × 10^6^) demonstrated significantly rapid growth, which could potentially interfere with the longitudinal therapeutic assessment. This determined the initial therapeutic planning for each approach, which mainly involved MHT. Furthermore, the histological tumor evaluation showed the presence of hypercellularity, a neoplastic epithelial population with marked pleomorphism, a large necrotic site, and a high number of mitotic cells (Figure 6II) on the 28th day after induction, common characteristics associated with breast cancer [89]. Therefore, before analysis, we opted for tumor induction with an intermediate concentration of 2 × 10^6^ cells and started MHT with a tumor size that was suitable for therapy and respected the clinical status of the animal for a longitudinal therapeutic follow-up of another week (from 14 to 21 days after induction).

The novel aspect of this study is demonstrated by the fact that, once the optimal conditions of the tumor model were established, each of the isolated and combined antitumor therapeutic approaches (exercise, omega-3, and MHT) was assessed to confirm their efficacy in treating and preventing triple-negative breast cancer. This combination has not been reported as a therapeutic approach in the literature to date. Only one study reported the antitumoral efficacy of combining a DHA with MHT supplementation (44 °C for 20 min) in HeLa and U937 tumor cells due to excess intracellular oxidative stress. The consequent activation of PKC-δ plays an important role in the enhancement of apoptosis, so cells treated with combined hyperthermia–DHA revealed significantly increased PKC-δ phosphorylation, leading to mitochondrial dysfunction and apoptosis in a DHA-dose-dependent manner [90].

Preclinical studies on breast cancer analyzing the isolated effect of each therapy, as proposed in this study, are well explored in the literature; however, there is still a wide variation in therapeutic parameters, such as the type and time of exercise [91], the dose, the route and type of omega-3 [43,54], and the MHT parameters (temperature, time, magnetic field, and oscillation frequency), as well as the type of magnetic nanoparticle used [92].

Considering the therapeutic approaches analyzed in this study, the transversal analysis (14 days after tumor induction; Figure 7 and Figure 8A,B), focusing on the effect of exercise and omega-3 supplementation, alone and combined, for 8 weeks, showed a significant difference in delays in tumor growth compared to the control (without any treatment), as shown in the BLI image. Interestingly, when comparing the results of exercise and omega-3, we found that the combined action was the most effective and the use of only omega-3 led to results that were very close to the combined therapy. This effect is little explored in the preclinical literature regarding the tumor reduction effect; however, in clinical studies, the reduction in the risk of cancer is evident [42,43], possibly due to the reduction in systemic pain, prevention of cachexia–anorexia, increased weight gain, reduced inflammatory response, and the side effects of chemotherapy [93], which also inhibit proliferation, preventing the formation of new blood vessels [43,94] and, consequently, metastasis [95], in addition to improving immune response [95,96,97].

In a longitudinal analysis of the efficacy of different therapeutic approaches (Figure 7 and Figure 8A,D), it was possible to verify a significant difference between the conditions before and after MHT therapy in all experimental groups associated with MTH (G2, G4, G6, and G8) versus the groups that were not associated with MTH (G1, G3, G5, and G7), in which it is evident that the use of MHT only led to a significant reduction in BLI signal, but when associated with each adjuvant therapy (omega-3 or physical training plus MTH; G6 and G4, respectively) the results were comparable to the use of MHT only. The combination of all three therapies (omega-3, plus physical training, plus MTH-G8) showed the best result, with a greater tumor reduction being evident (81.2% of tumor signal decay) when analyzed indirectly by the BLI, representing a pioneering and promising therapeutic proposal for the treatment of breast cancer that is not evident in the literature to date.

When analyzing only groups that received MHT, we observed that the combination of MHT and exercise and omega-3 supplementation also led to tumor reductions, but without any statistical difference. Therefore, the combination of exercise and omega-3 showed the potential to slow tumor growth before MTH therapy and to increase the efficacy of MTH.

However, even though promising results were obtained regarding tumor reduction when evaluated by BLI, the combined therapy that was studied should be better explored, focusing on other aspects and in a more specific way, to better understand the molecular, genetic, and clinical impact in the long term, and to verify metastases and recurrences.

## 5. Conclusions

The pioneering therapeutic approach proposed in this study showed positive implications for the treatment of breast cancer after establishing a suitable model, and the results indicated that MHT plays a significant role in reducing tumor size and has greater effectiveness when combined with adjuvant treatments like omega-3 and exercise. These results have the potential to direct future pre-clinical and clinical research on the development of more effective and personalized therapeutic approaches to treatment for breast cancer, as well as other illnesses.

## Figures and Tables

**Table 1:** The experimental groups used to assess the efficacy of the combination of magneto-hyperthermia therapy, omega-3 supplementation, and physical training.

Condition	G1 (n = 8)	G2 (n = 8)	G3 (n = 8)	G4 (n = 8)	G5 (n = 8)	G6 (n = 8)	G7 (n = 8)	G8 (n = 8)
Tumor Induction	●	●	●	●	●	●	●	●
Physical Training			●	●			●	●
ω-3 supplementation					●	●	●	●
MHT		●		●		●		●

Abbreviations: MHT: magneto-hyperthermia; ω-3: omega-3. Note: G1 (control); G2 (only MHT); G3 (only physical training); G4 (physical training + MHT); G5 (only omega-3); G6 (omega-3 + MHT); G7 (physical training+ omega-3); G8 (physical training+ omega-3 + MHT).

**Table 2:** Post hoc analysis between groups from the in vitro magneto-hyperthermia experiment.

Comparisons		Mean Difference	SE	*t*	*p*
MDA-MB-231 + AMF	MDA-MB-231 + ION	3.603 × 10^7^	6.749 × 10^6^	5.338	0.001
	MDA-MB-231 + ION + AMF	1.291 × 10^9^	6.749 × 10^6^	191.339	<0.001
MDA-MB-231 + ION	MDA-MB-231 + ION + AMF	1.255 × 10^9^	6.749 × 10^6^	186.001	<0.001

Abbreviations: SE: standard error; AMF: alternating magnetic field; ION: iron–oxide nanoparticles.

**Table 3:** Description of the intensity of the bioluminescence signal with the mean and standard deviation of the experimental groups before (after 14 days of tumor induction) and after in vivo magneto-hyperthermia (after 21 days of tumor induction).

Groups	Before MHT (14D)		After MHT (21D)		Statistic
	Mean (Photon/s)	SD	Mean (Photon/s)	SD	*p*
G1	3.524 × 10^9^	3.155 × 10^8^	4.508 × 10^9^	4.082 × 10^8^	<0.001
G2			9.228 × 10^8^	3.279 × 10^7^	<0.001
G3	2.785 × 10^9^	6.769 × 10^8^	3.000 × 10^9^	5.729 × 10^8^	0.816
G4			6.363 × 10^8^	1.498 × 10^8^	<0.001
G5	2.055 × 10^9^	5.100 × 10^8^	2.723 × 10^9^	5.160 × 10^8^	0.074
G6			2.995 × 10^8^	7.784 × 10^7^	<0.001
G7	1.961 × 10^9^	3.495 × 10^8^	2.313 × 10^9^	3.495 × 10^8^	0.116
G8			1.520 × 10^8^	3.080 × 10^7^	<0.001

Abbreviations: MHT: magneto-hyperthermia; SD: standard deviation. Note: G1 (control); G2 (only MHT); G3 (only physical training); G4 (physical training + MHT); G5 (only omega-3); G6 (omega-3 + MHT); G7 (physical training + omega-3); G8 (physical training + omega-3 + MHT).

**Table 4:** Post hoc analysis between experimental groups of the bioluminescence signal intensity before application of the in vivo magneto-hyperthermia therapy.

Comparisons		Mean Difference	SE	*t*	*p*
G1 + G2-14D	G3 + G4-14D	7.387 × 10^8^	2.424 × 10^8^	3.048	0.030
	G5 + G6-14D	1.469 × 10^9^	2.424 × 10^8^	6.059	<0.001
	G7 + G8-14D	1.563 × 10^9^	2.424 × 10^8^	6.446	<0.001
G3 + G4-14D	G5 + G6-14D	7.300 × 10^8^	2.424 × 10^8^	3.012	0.033
	G7 + G8-14D	8.238 × 10^8^	2.424 × 10^8^	3.398	0.012
G5 + G6-14D	G7 + G8-14D	9.375 × 10^7^	2.424 × 10^8^	0.387	1.000

Abbreviations: SE: standard error; D: day. Note: G1 + G2 (control), G3 + G4 (only physical training), G5 + G6 (only omega-3), and G7 + G8 (physical training + omega-3).

**Table 5:** Post hoc test for two-by-two comparison within groups before and after the application of in vivo MTH.

Comparisons		Mean Difference	SE	*t*	*p*
G1 + G2-14D	G1-D21	−9.837 × 10^8^	1.860 × 10^8^	−5.288	<0.001
	G2-D21	2.601 × 10^9^	1.860 × 10^8^	13.981	<0.001
G1-D21	G2-D21	3.585 × 10^9^	2.148 × 10^8^	16.687	<0.001
G3 + G4-D14	G3-D21	−2.150 × 10^8^	3.507 × 10^8^	−0.613	0.816
	G4-D21	2.149 × 10^9^	3.507 × 10^8^	6.127	<0.001
G3-D21	G4-D21	2.364 × 10^9^	4.049 × 10^8^	5.837	<0.001
G5 + G6-D14	G5-D21	−6.675 × 10^8^	2.758 × 10^8^	−2.420	0.074
	G6-D21	1.756 × 10^9^	2.758 × 10^8^	6.365	<0.001
G5-D21	G6-D21	2.423 × 10^9^	3.185 × 10^8^	7.608	<0.001
G7 + G8-D14	G7-D21	−3.512 × 10^8^	1.624 × 10^8^	−2.163	0.116
	G8-D21	1.809 × 10^9^	1.624 × 10^8^	11.141	<0.001
G7-D21	G8-D21	2.161 × 10^9^	1.875 × 10^8^	11.522	<0.001

Abbreviations: SE: standard error; D: day. Note: G1 + G2 (control), G3 + G4 (only physical training), G5 + G6 (only omega-3), G7 + G8 (physical training + omega-3), G1 (control), G2 (only MHT), G3 (only physical training), G4 (physical training + MHT), G5 (only omega-3), G6 (omega-3 + MHT), G7 (physical training + omega-3), and G8 (physical training + omega-3 + MHT).

**Table 6:** Post hoc test for two-by-two comparison between groups after the application of in vivo MTH.

Comparisons		Mean Difference	SE	*t*	*p*
G2_D21	G4-D21	2.865 × 10^8^	6.178 × 10^7^	4.637	0.003
	G6-D21	6.232 × 10^8^	6.178 × 10^7^	10.088	<0.001
	G8-D21	7.707 × 10^8^	6.178 × 10^7^	12.476	<0.001
G4-D21	G6-D21	3.368 × 10^8^	6.178 × 10^7^	5.451	<0.001
	G8-D21	4.842 × 10^8^	6.178 × 10^7^	7.838	<0.001
G6-D21	G8-D21	1.475 × 10^8^	6.178 × 10^7^	2.388	0.133

Abbreviations: SE: standard error; D: day. Note: G1 (control); G2 (only MHT); G3 (only physical training); G4 (physical training + MHT); G5 (only omega-3); G6 (omega-3 + MHT); G7 (physical training + omega-3); G8 (physical training + omega-3 + MHT).

**Table 7:** Description of the percentage effectiveness of magneto-hyperthermia therapy, showing the mean and standard deviation between groups. A positive value indicates tumor growth and a negative value indicates decreased tumor size.

Groups	Efficacy (%)	
	Mean	SD
G1	+30.3	1.8
G2	−74.1	2.6
G3	+29.0	1.1
G4	−78.1	4.8
G5	+18.7	6.8
G6	−82.8	5.3
G7	+13.7	18.0
G8	−91.2	3.8

Abbreviations: SD: standard deviation. Note: G1 (control). G2 (only MHT); G3 (only physical training); G4 (physical training + MHT); G5 (only omega-3); G6 (omega-3 + MHT); G7 (physical training + omega-3); G8 (physical training + omega-3 + MHT).

## Data Availability

All data analyzed during this study are included in this manuscript.
